# Access to and use of high blood pressure medications in Brazil

**DOI:** 10.1590/S1518-8787.2016050006154

**Published:** 2016-12-01

**Authors:** Sotero Serrate Mengue, Andréa Dâmaso Bertoldi, Luiz Roberto Ramos, Mareni Rocha Farias, Maria Auxiliadora Oliveira, Noemia Urruth Leão Tavares, Paulo Sergio Dourado Arrais, Vera Lucia Luiza, Tatiane da Silva Dal Pizzol

**Affiliations:** I Programa de Pós-Graduação em Epidemiologia. Faculdade de Medicina. Universidade Federal do Rio Grande do Sul. Porto Alegre, RS, Brasil; II Departamento de Medicina Social. Faculdade de Medicina. Universidade Federal de Pelotas. Pelotas, RS, Brasil; III Departamento de Medicina Preventiva. Escola Paulista de Medicina. Universidade Federal de São Paulo. São Paulo, SP, Brasil; IV Departamento de Ciências Farmacêuticas. Centro de Ciências da Saúde. Universidade Federal de Santa Catarina. Florianópolis, SC, Brasil; V Departamento de Política de Medicamentos e Assistência Farmacêutica. Escola Nacional de Saúde Pública Sérgio Arouca. Fundação Oswaldo Cruz. Rio de Janeiro, RJ, Brasil; VI Departamento de Farmácia. Faculdade de Ciências da Saúde. Universidade de Brasília. Brasília, DF, Brasil; VII Departamento de Farmácia. Faculdade de Farmácia, Odontologia e Enfermagem. Universidade Federal do Ceará. Fortaleza, CE, Brasil; VIII Departamento de Produção e Controle de Medicamentos. Faculdade de Farmácia. Universidade Federal do Rio Grande do Sul. Porto Alegre, RS, Brasil

**Keywords:** Antihypertensive Agents, supply & distribution, Drug Utilization, Drugs of Continuous Use, Health Services Accessibility, Socioeconomic Factors, Health Surveys

## Abstract

**OBJECTIVE:**

To analyze the access to and use of medicines for high blood pressure among the Brazilian population according to social and demographic conditions.

**METHODS:**

Analysis of data from *Pesquisa Nacional Sobre Acesso, Utilização e Promoção do Uso Racional de Medicamentos* (PNAUM – National Survey on Access, Use and Promotion of Rational Use of Medicines), a nationwide cross-sectional, population-based study, with probability sampling, carried out between September 2013 and February 2014 in urban households in the five Brazilian regions. The study evaluated the access and use of medicines to treat people with high blood pressure. The independent variables were gender, age, socioeconomic status and Brazilian region. The study also described the most commonly used drugs and the percentage of people treated with one, two, three or more drugs. Point estimations and confidence intervals were calculated considering the sample weights and sample complex plan.

**RESULTS:**

Prevalence of high blood pressure was 23.7% (95%CI 22.8–24.6). Regarding people with this condition, 93.8% (95%CI 92.8–94.8) had indication for drug therapy and, of those, 94.6% (95%CI 93.5–95.5) were using the medication at the time of interview. Full access to medicines was 97.9% (95%CI 97.3–98.4); partial access, 1.9% (95%CI 1.4–2.4); and no access, 0.2% (95%CI 0.1–0.4). The medication used to treat high blood pressure, 56.0% (95%CI 52.6–59.2) were obtained from SUS (Brazilian Unified Health System), 16.0% (95%CI 14.3–17.9) from Popular Pharmacy Program, 25.7% (95%CI 23.4–28.2) were paid for by the patients themselves and 2.3% (95%CI 1.8–2.9) were obtained from other locations. The five most commonly used drugs were, in descending order, hydrochlorothiazide, losartan, captopril, enalapril and atenolol. Of the total number of patients on treatment, 36.1% (95%CI 34.1–37.1) were using two medicines and 13.5% (95%CI 12.3–14.9) used three or more.

**CONCLUSIONS:**

Access to medicines for the treatment of high blood pressure may be considered high and many of them are available free of charge. The most commonly used drugs are among those recommended as first-line treatment for high blood pressure control. The percentage of people using more than one drug seems to follow the behavior observed in other countries.

## RESUMO

**OBJETIVO:**

Analisar o acesso e a utilização de medicamentos para a hipertensão na população brasileira segundo condições sociais e demográficas. Análise dos dados da Pesquisa Nacional Sobre Acesso, Utilização e Promoção do Uso Racional de Medicamentos, estudo nacional de delineamento transversal de base populacional, com amostra probabilística, realizado entre setembro de 2013 e fevereiro de 2014 em domicílios urbanos nas cinco regiões do Brasil. Avaliou-se o uso e acesso aos medicamentos para os cuidados com pessoas que apresentam hipertensão arterial. As variáveis independentes utilizadas foram sexo, idade, nível socioeconômico e região do País. Também foram descritos os fármacos mais utilizados e a proporção de pessoas tratadas com um, dois, três ou mais fármacos. As estimativas de ponto e os intervalos de confiança foram calculados considerando os pesos amostrais e o plano complexo da amostra. A prevalência de hipertensão arterial foi de 23,7% (IC95% 22,8–24,6). Das pessoas com a condição, 93,8% (IC95% 92,8–94,8) tinham indicação de tratamento com medicamentos e, destes, 94,6% (IC95% 93,5–95,5) estavam usando os medicamentos no momento da entrevista. O acesso total aos medicamentos foi de 97,9% (IC95% 97,3–98,4); o acesso parcial, de 1,9% (IC95% 1,4–2,4); e o acesso nulo, de 0,2% (IC95% 0,1–0,4). Dos medicamentos utilizados para tratar a hipertensão, 56,0% (IC95% 52,6–59,2) foram obtidos no SUS, 16,0% (IC95% 14,3–17,9), no Programa Farmácia Popular, 25,7% (IC95% 23,4–28,2) pago do próprio bolso e 2,3% (IC95% 1,8–2,9) em outros locais. Os cinco fármacos mais utilizados foram, em ordem descrente, hidroclorotiazida, losartana, captopril, enalapril e atenolol. Do total de tratados, 36,1% (IC95% 34,1–37,1) estavam usando dois fármacos e 13,5% (IC95% 12,3–14,9) utilizavam três ou mais fármacos.

**CONCLUSÕES:**

**:** O acesso aos medicamentos para tratamento da hipertensão pode ser considerado elevado e grande parte desses medicamentos é obtida gratuitamente. Os fármacos mais utilizados estão entre os preconizados como de primeira linha para o controle de hipertensão arterial. A proporção de pessoas utilizando mais de um fármaco parece seguir o comportamento observado em outros países.

## INTRODUCTION

Non-communicable diseases (NCD) are a global health problem and have influenced worldwide initiatives aimed to their control and reduction^1^. In Brazil, NCD represent a high percentage of diseases, accounting for much of the mortality and the higher cost of hospitalizations, especially among older adults^21^.

Schmidt et al.^31^ report that despite the increase in crude mortality rates in recent years, age-specific mortality rates have decreased. According to the author, this was due to the success of a number of public policies to reduce tobacco use and expand access to basic health care. Prominent among these public policies are *Plano de Reorganização da Atenção ao Diabetes e Hipertensão* ^a^ (Plan for the Reorganization of Care for Arterial Hypertension and Diabetes Mellitus, *Política Nacional de Medicamentos* ^18^ (National Medicine Policy), *Política Nacional de Assistência Farmacêutica* ^b^ (National Policy on Pharmaceutical Services) and *Plano Nacional de Enfrentamento das DCNT* (Brazilian Plan to Combat NCD) in Brazil for the period 2011-2022^c^.

Among NCD, high blood pressure accounts for most of the patients^31^. High blood pressure is a treatable medical condition and, if properly controlled, can delay or even prevent the development of symptomatic cardiovascular disease^23^. Medications play an important therapeutic role in hypertension care, both for their low cost in the early stages of the disease and for adherence to treatment, which, up to now, has been higher than adherence to lifestyle changes^12^. The global scenario of NCD led the World Health Organization to launch, in 2012, the challenge to reduce mortality from these diseases by 25.0% by 2052^2^. The WHO considered access to medicines an essential component in achieving this goal^2^ ^,^ ^6^.

In Brazil, basic health care policies, including the expansion of free access to drugs, are considered examples of public initiatives^3^ and control of NCD. The objective of this study was to analyze the access to and use of medicines for high blood pressure among the Brazilian population, according to social and demographic conditions.

## METHODS

The data for this analysis is taken from PNAUM (National Survey on Access, Use and Promotion of Rational Use of Medicines), a cross-sectional, population-based study. The study population of PNAUM consisted of people living in permanent private households in urban areas of the five Brazilian regions. The study analyzed data of people aged 20 or older. The questionnaires were answered directly by the person or by a caregiver, when they were unable to communicate or provide information on diseases and medicines used.

The sampling plan was complex and resulted in a probability sampling of the Brazilian population residing in urban areas of Brazil. Information on the sample, sampling procedures and other methodological procedures of PNAUM, including instruments used in the interview, field operation aspects and specificities of data collection are available in the PNAUM methodological article^17^.

The use of medicines to treat high blood pressure was investigated based on the following questions: “Has a doctor ever said you have hypertension or high blood pressure?”; “Do you have any medical indication to take any kind of medicine for high blood pressure?”; “Are you taking any of those medicines?”. Later, they were asked about the drugs being used for high blood pressure and detailed information on source and payment, among others aspects. Whenever possible, the drug names were copied from the packaging. When no packaging was available, the names declared by the interviewees were recorded.

The sociodemographic characteristics analyzed were: gender, age group (in two distinct variables: 20-39, 40-59 and 60 or over, and 20-59 and 60 or over), economic status according to *Associação Brasileira de Empresas de Pesquisa* (ABEP – Brazilian Association of Survey Companies)^d^ (A/B, C, D/E) and Brazilian region (North, Northeast, Southeast, South, Midwest). The denominator in calculating prevalence of use was the total number of people aged 20 or over in the sample, with 95% confidence intervals (95%CI).

The access to medicines was classified as full, partial or no access. Full access was the situation in which people had all medicines needed to care for the disease in the last 30 days; partial, when any medicine was missing due to financial difficulty or unavailability at the Unified Health System (SUS); and no access, when no drugs were obtained in the last 30 days, also due to financial difficulty or unavailability at SUS. The denominator comprised people with medical indication to treat high blood pressure with drugs, excluding cases of intentional abandonment of treatment. Abandonment was defined as cases in which, despite medical indication, people were not using medications for high blood pressure; and cases in which they declared to be using the drugs, but had been without in the last 30 days. In both situations, abandonment cases were restricted to lack of medicines for reasons other than financial difficulty or unavailability at SUS.

Reported medicines for high blood pressure were classified into four groups, according to the reported use for treating high blood pressure. Group 1 consisted of drugs definitely used for high blood pressure, such as diuretics, angiotensin-converting enzyme inhibitors and angiotensin receptor blockers. Group 2 included drugs associated with patients’ care with high blood pressure, such as statins and acetylsalicylic acid. Group 3 corresponded to medicines unrelated to high blood pressure treatment, but reported by respondents as used for that disease. Group 4 comprised all teas or other supplements not classified as medicines. This classification was based on high blood pressure treatment guidelines and a review of the literature for some controversial cases in group 2.

The overall results of this analysis include all medications. The drugs analysis relates exclusively to drugs specifically used for high blood pressure treatment (group 1).

In the analysis of drugs used for high blood pressure treatment, fixed-dose combinations were broken down into isolated drugs. For example, for people who used a medicine with a fixed combination of hydrochlorothiazide and amiloride, each one of those drugs was analyzed separately in the evaluations that considered the number of specific drugs for the treatment of high blood pressure.

Pearson’s Chi-square test was used for percentage bivariate comparison, considering p < 0.05 as significant values.

This study was approved by the *Comissão Nacional de Ética em Pesquisa* (CONEP – National Research Ethics Commission) and is registered under number 18947013.6.0000.0008. All interviews were conducted after the respondents or their legal representatives had read and signed the consent form.

## RESULTS

Prevalence of self-reported high blood pressure in Brazil was 23.7% (95%CI 22.8–24.6), ranging from 16.3% in the North region to 26.0% in the Southeast region. In the 20-39 age group, prevalence was 6.0%, reaching 59.0% in the 60 or over group (Table 1).

**Table 1:** Prevalence of self-reported high blood pressure, indication of treatment and use of medication for high blood pressure among the Brazilian population aged 20 or over, according to sociodemographic characteristics, PNAUM, Brazil, 2104a.

Variable		Prevalence of hypertension			Hypertension patients with indication for drug treatment			Hypertension patients with indication for drug treatment using medication		p
		
		%	95%CI	p	%	95%CI	p	%	95%CI	
Gender	Male	18.5	17.4–19.8		93.1	91.2–94.6		93.4	91.5–94.9	
	Female	28.1	27.0–29.1	< 0.001	94.1	92.9–95.1	0.292	95.2	94.2–96.1	0.026
Age group	20 to 39	6.0	5.2–6.9		78.0	71.1–83.6		78.4	70.8–84.5	
	40 to 59	27.5	26.3–28.8		93.4	91.9–94.7		94.7	93.4–95.8	
	60 or over	59.0	57.4–60.5	< 0.001	98.1	97.6–98.5	< 0.001	97.6	97.0–98.1	< 0.001
Brazilian region	North	16.3	14.6–18.1		91.7	89.0–93.9		91.4	88.1–93.8	
	Northeast	21.7	20.3–23.1		92.2	90.0–93.9		91.1	87.6–93.7	
	Southeast	26.0	24.3–27.7		94.9	93.0–96.2		95.6	94.2–96.7	
	South	22.4	20.8–24.0		93.0	90.4–95.0		97.4	96.1–98.2	
	Midwest	24.1	22.0–26.3	< 0.001	92.9	90.4–94.8	0.495	94.0	92.2–95.5	< 0.001
CCEB^b^	A/B	22.3	20.4–24.3		94.7	92.6–96.2		96.2	94.3–97.5	
	C	23.9	22.8–25.0		93.4	91.9–94.7		94.8	93.6–95.8	
	D/E	24.7	23.1–26.3	0.127	93.5	91.5–95.1	0.064	92.3	89.2–94.6	0.019

Total		23.7	22.8–24.6		93.7	92.6–94.7		94.6	93.5–95.5	

^a^ Percentage adjusted by sample weights and post-stratification according to age and gender.

^b^ Classified according to *Critério de Classificação Econômica Brasil 2013* (CCEB 2013 – Brazilian Economic Classification Criterion) of *Associação Brasileira de Empresas de Pesquisa* (ABEP – Brazilian Association of Survey Companies). Available from: http://www.abep.org

Of the total number of people who reported high blood pressure, 93.8% affirmed having medical indication to treat the disease with medication. This indication ranged from 78.0% in the 20-39 age group to 98.1% in the group aged 60 or over (Table 1).

Among people who reported having indication for drug therapy, 94.6% were using the medication at the time of the interview. Use of medication increased with age. Among Brazilian regions, the North and Northeast regions had the lowest frequency of drug use, while the South and Southeast regions had the highest frequency of use (Table 1).

Full access to medicines for high blood pressure care was higher in the South region and lower in the Midwest and Northeast regions. Partial access, i.e., lack of part of the drugs in the previous 30 days, was higher in the Midwest region and lower in the South region. Prevalence of access to all necessary medicines for high blood pressure care was similar between the different economic classes. Failure to obtain any hypertension medication for financial difficulties or unavailability at SUS was very low throughout the country, with minor, non-significant differences between the age groups (Table 2). Intentional abandonment of treatment reached 10.6% of cases.

**Table 2:** Access to high blood pressure medication by hypertension patients with medical indication for drug treatment by age, gender, ABEP and Brazilian region. PNAUM, Brazil, 2104a.

Variable		Full access^b^		Partial access^c^		No access^d^		p
		
		%	95CI%	%	95CI%	%	95CI%	
Gender	Male	98.6	97.8–99.0	1.3	0.9–2.0	0.1	0.0–0.3	
	Female	97.6	96.8–98.2	2.2	1.6–3.0	0.3	0.1–0.5	0.046
Age group	20 to 59	97.2	96.1–97.9	2.5	1.7–3.5	0.4	0.2–0.7	
	60 or over	98.6	98.0–99.1	1.3	0.9–1.9	0.05	0.0–0.1	0.001
Brazilian region	North	97.1	93.6–98.7	2.5	1.0–6.2	0.3	0.2–0.7	
	Northeast	96.1	94.4–97.2	3.3	2.2–4.9	0.6	0.3–1.4	
	Southeast	98.7	97.7–99.2	1.3	0.7–2.2	0.1	0.0–0.4	
	South	99.2	98.3–99.6	0.7	0.3–1.6	0.1	0.0–0.5	
	Midwest	95.7	93.8–97.0	4.0	2.7–5.8	0.4	0.1–1.1	< 0.001
CCEB^e^	A/B	98.6	97.6–99.2	1.0	0.5–2.0	0.4	0.1–1.2	
	C	97.7	96.8–98.3	2.2	1.6–3.0	0.1	0.1–0.3	
	D/E	97.7	96.6–98.5	2.0	1.3–3.2	0.3	0.1–0.6	0.103

Total		97.9	97.3–98.4	1.9	1.4–2.4	0.2	0.1–0.4	

^a^ Percentage adjusted by sample weights and post-stratification according to age and gender.

^b^ Full access: provision of all necessary medicines to treat hypertension in the previous 30 days, either free of charge or paid for by patients.

^c^ Lack of any medicine to treat hypertension in the previous 30 days due to financial difficulties or unavailability at SUS (Brazilian Unified Health System).

^d^ No access due to financial difficulties or unavailability at SUS.

^e^ Classified according to *Critério de Classificação Econômica Brasil 2013* (CCEB 2013 – Brazilian Economic Classification Criterion) of *Associação Brasileira de Empresas de Pesquisa* (ABEP – Brazilian Association of Survey Companies). Available from: http://www.abep.org

Of all the medicines reported for use in high blood pressure, group 1 (medicines definitely used to treat high blood pressure) corresponded to 90.5%, group 2 (drugs associated with high blood pressure treatment), to 4.1%, group 3 (drugs not indicated for high blood pressure), to 3.2%, and group 4 (supplements and herbs), to 2.3%. Of medicines used specifically to treat high blood pressure (group 1), 92.4% were single-drug and 7.6% were fixed-dose combinations, the most common being hydrochlorothiazide combined with losartan or atenolol or amiloride. Separately, the most common specific drugs used to treat high blood pressure were, in descending order: hydrochlorothiazide, losartan, captopril, enalapril, amlodipine and atenolol, which corresponded to approximately 81.0% of all reported drugs (Table 3).

**Table 3:** Most commonly used medicines to treat high blood pressure, regardless of fixed-dose combinations or number of drugs used. PNAUM, Brazil, 2014*.

Drug	%	95CI%	cumulative %
Hydrochlorothiazide	23.9	22.7–25.1	23.9
Losartan	20.1	19.0–21.3	44.0
Captopril	11.2	10.2–12.2	55.2
Enalapril	10.5	9.4–11.7	65.7
Atenolol	9.0	8.2–9.9	74.6
Amlodipine	6.0	5.4–6.6	80.6
Propranolol	4.1	3.6–4.7	84.7
Furosemide	2.4	2.0–2.8	87.1
Nifedipine	1.8	1.5–2.2	88.9
Chlorthalidone	1.8	1.4–2.2	90.7
Other hypertension drugs	9.3	8.4–10.3	100

* Percentage adjusted by sample weights and post-stratification according to age and gender.

Among respondents treated with specific drugs for hypertension, 49.9% used only one drug, 36.1% used two, and 13.5% used three or more drugs (Table 4). High blood pressure treatment with more than one drug increased with age. In the 20 to 39 age group, 43.3% of hypertensive patients used more than one drug, while for those over 60 the figure was 52.2%. Differences were also observed among individuals suffering only from high blood pressure. Of those, 46.0% treated hypertension with more than one drug, and among people with two or more other chronic diseases, 55.8% used more than one drug. In the North region, the use of two or more drugs was lower than that observed in the other Brazilian regions.

**Table 4:** Percentage of number of drugs, regardless of fixed-dose combinations, used to treat high blood pressure (HBP) per age group, number of chronic diseases and Brazilian region. PNAUM, Brazil, 2014*.

Variable		One drug	95CI%	Two drugs	95CI%	Three or more drugs	95CI%	p
Gender	Male	52.4	46.9–55.2	35.5	32.9–38.2	12.0	10.3–14.0	0.034
	Female	48.5	46.6–50.5	37.2	35.4–39.0	14.3	12.9–15.9	
Age group	20 to 39	56.7	49.0–64.1	33.1	25.6–41.5	10.2	5.9–17.0	0.061
	40 to 59	51.1	48.2–49.8	36.9	34.1–39.8	12.0	10.3–13.8	
	60 or over	47.8	45.9–49.8	36.9	35.1–38.7	15.3	13.7–17.0	
Number of chronic diseases	HBP only	54.0	51.3–56.6	36.3	34.0–38.6	9.8	8.0–11.9	0.001
	HBP and another chronic disease	51.8	48.8–54.9	35.4	32.5–38.4	12.8	10.9–14.9	
	HBP and two or more other chronic diseases	44.1	41.5–46.9	37.8	35.5–40.3	18.0	15.9–20.2	
Brazilian region	North	71.9	68.6–75.0	24.7	22.0–27.6	3.4	2.6–4.6	0.001
	Northeast	48.7	45.7–51.6	38.6	35.6–41.5	12.7	10.8–14.8	
	Southeast	48.5	46.0–51.1	36.9	34.6–39.3	14.6	12.6–16.9	
	South	51.7	48.0–55.3	35.8	32.9–38.8	12.5	10.7–14.5	
	Midwest	47.5	43.7–51.3	37.1	33.9–40.4	15.5	13.1–18.1	

Total		49.9	48.3–51.5	36.1	34.1–37.1	13.5	12.3–14.9	

* Percentage adjusted by sample weights and post-stratification according to age and gender.

Of medicines used to treat high blood pressure, 56.0% were obtained from SUS, 16.0% from *Programa Farmácia Popular* (program-specific or accredited drugstores) and 2.3% elsewhere. Payment by the actual patients corresponded to 25.7% of medications used to treat this condition.

## DISCUSSION

Prevalence of self-reported high blood pressure in this study was 23.7% for all ages, 18.5 in men and 28.1 in women. Barros et al.^5^, analyzing data from the 2008 *Pesquisa Nacional por Amostra de Domicílios* (PNAD – National Household Sample Survey), found a prevalence of 11.3% for men, 16.5% for women and 14.0% for both genders and all ages. In a summary measure of several studies, Schmidt et al.^31^ found a 17.3% prevalence for men and women over 20. Among men, the prevalence of 19.2% was slightly higher than in the 2008 survey, when it was estimated at 17.3% (95%CI 17.0–17.6%). In the VIGITEL telephone survey^e^ for all 27 cities, considering adults (aged over 18), prevalence was 24.1%, with a higher rate of high blood pressure among women (26.3%) than men (21.5 %). The 2013 *Pesquisa Nacional de Saúde* (National Health Research)^f^ showed a prevalence of self-reported hypertension of 21.4% (95%CI 20.8–22.0) for people over 18.

When compared with previous population studies, prevalence of self-reported high blood pressure in this study confirms the upward trend of this disease in all age groups. The figures found in the Vigitel survey are higher, probably because this tool included only state capitals and metropolitan areas of Brazil, where prevalence of hypertension tends to be higher, whether due to increased presence of risk factors, greater access to diagnosis or a combination of both factors.

In this study, the question on the use of medicines for high blood pressure was preceded by another question about the existence of medical indication for treatment of high blood pressure with medications. This question was included after the study identified that one of the reasons participants were not using drugs was the lack of medical indication.

Ferreira et al.^11^, analyzing data of the 2008 PNAD^25^, found that 17.0% of patients did not use high blood pressure medication. The study shows that of people with hypertension, 6.2% had no medical indication to treat the disease with medication, and only 5.4% of those with indication were not using the drugs. The difference in the results of both studies may be due the authors assuming that all patients with high blood pressure should use medicines to treat the condition.

Indication for treatment with medication is high. This may reflect late diagnosis, given that in stage 1 hypertension, lifestyle change is the first indication, with drug treatment only introduced six months after that measure fails^g^. When diagnosis is delayed, greater severity of the disease or less success in the use of non-drug treatment is expected, due to lack of lifestyle changes. A further aspect is the behavior of doctors, who, expecting that patients will not adhere to non-drug treatment, introduce drug treatment alongside lifestyle change recommendations.

Analysis of PNAD^h^ data showed a significant difference in drug use between men and women, with lower rates among men. Data from this study show a slightly lower use among men compared to women and this difference was not significant.

Also consistent are the lower rates of medicine use among younger age groups. This study shows that younger people receive less indication for drug treatment, and when they do, adherence is very low compared to older people. Consequently, young people miss the opportunity to prematurely reduce cardiovascular risks with the use of medicines and may experience further harm in future complications of the clinical condition.

Overall, indication and use of medications were high, which should be considered positive, especially because it is an asymptomatic clinical condition. However, a more accurate assessment of treatment adequacy and follow-up is needed as a next step in the care of hypertension. Evidence shows that inadequate understanding of the nature of hypertension leads to discontinuation of treatment when blood pressure levels are normalized. Other causes for temporary interruption or undertreatment of the disease are the occurrence of side effects, forgetfulness, or reasons related to health services, such as organization and structure, sometimes involving the relationship between doctor and patient^2^ ^,^ ^9^.

Nationwide data on access to medicines are still scarce, especially at the household level. Paniz et al.^19^, studying users in the program *Estratégia de Saúde da Família* (Family Health Strategy) in 41 municipalities in the South and Northeast regions in 2005, found that 81.2% had full access, albeit with regional differences, rates being higher in the South. Increased age, better socioeconomic status and higher education level were factors associated with greater access. In one state capital in southern Brazil, access was 95.8%^5^. Among the population covered by the *Programa de Saúde da Família* (Family Health Program) in another state capital in southern Brazil, overall access to medicines was also 95.0%^8^. Those studies show high access to medicines for high blood pressure, and, on average, half of the medications was obtained free of charge from SUS. This study shows an increase in drugs obtained from the SUS basic network, as well as from the Farmácia *Popular* (Popular Pharmacy Program), which accounted for 16.0% of medicines used in hypertension care.

Most part of the medicines used in hypertension care is available for free, whether from the SUS network or drugstores accredited by the program *Aqui Tem Farmácia Popular* (Popular Pharmacy Here). Thus, almost three out of four medicines used in high blood pressure care were funded by SUS.

Of the 10 drugs most commonly used to treat hypertension, only chlorthalidone did not feature in *Relação Nacional de Medicamentos Essenciais* (Rename – National List of Essential Medicines) for 2013^i^. Of those 10 drugs, nine can be obtained from the SUS basic network and Popular Pharmacy Program network free of charge. Moreover, municipal lists of medications may include those drugs, making them available to patients through SUS in such municipalities.

The most commonly used drugs were hydrochlorothiazide, followed by losartan, captopril and enalapril. These results are similar to those found in other studies in which diuretics and renin-angiotensin system antagonists are the drugs used to treat high blood pressure^13^ ^,^ ^22^. A meta-analysis of 31 randomized controlled trials involving 190,606 participants carried out by the Blood Pressure Lowering Treatment Trialists’ Collaboration^8^ showed no significant differences between the various drugs used to treat high blood pressure in the prevention of cardiovascular events.

High blood pressure treatment with more than one drug is observed in other countries. In England, 45.0% of patients undertake monotherapy; 36.0%, two drugs; 14.0%, three drugs; and 4.0%, four drugs^10^. In the United States, the percentage of patients who use more than one drug rose from 29.1% in 1988-1994 to 35.8% in 1999-2002^14^ and to 47.7% in 2009-2010^15^.

In short, prevalence of high blood pressure continues to increase among the adult Brazilian population. Access to medicines to treat this disease proved to be quite high. Compared with previous studies, this increase appears to be consistent. Pharmaceutical care public policies may explain this phenomenon. Most of the drugs used are among those recommended in the literature as first-line drugs for high blood pressure treatment. In this study, the number of drugs used shows the same trend observed in the United States and England, i.e., a growth in the number of drugs needed to keep blood pressure under adequate control.

Full access to medicines for high blood pressure can be seen as the first step in the treatment of this clinical condition, but not the only one. About 50.0% of treated patients do not have adequate blood pressure control^16^ ^,^ ^20^. Access to drugs must be complemented by good adherence to treatment, appropriate management of the drugs for each patient, and control of other factors that may hinder blood pressure control, such as high salt intake or obstructive sleep apnea^21^.

## Footnotes

a Ministério da Saúde, Secretaria de Políticas de Saúde, Departamento de Ações Programáticas Estratégicas. Plano de reorganização da atenção à hipertensão arterial e ao diabetes mellitus. Brasília (DF); 2001. (Série C. Projetos, Programas e Relatórios, 59). Available from: http://bvsms.saude.gov.br/bvs/publicacoes/miolo2002.pdf

b Ministério da Saúde, Conselho Nacional de Saúde. Resolução nº 338 de 6 de maio de 2004. Aprova a Política Nacional de Assistência Farmacêutica. Brasília (DF); 2004. Available from: http://bvsms.saude.gov.br/bvs/saudelegis/cns/2004/res0338_06_05_2004.html

c Ministério da Saúde, Secretaria de Vigilância em Saúde, Departamento de Análise de Situação de Saúde. Plano de ações estratégicas para o enfrentamento das doenças crônicas não transmissíveis (DCNT) no Brasil 2011-2022. Brasília (DF); 2011. (Série B. Textos Básicos de Saúde). Available from: http://bvsms.saude.gov.br/bvs/publicacoes/plano_acoes_enfrent_dcnt_2011.pdf

d Associação Brasileira de Empresas de Pesquisa. Critério de Classificação Econômica Brasil 2013. São Paulo (SP): ABEP; 2013. Available from: http://www.abep.org/criterio-brasil

e Ministério da Saúde, Secretaria de Vigilância em Saúde. Vigitel Brasil 2013: vigilância de fatores de risco e proteção para doenças crônicas por inquérito telefônico. Brasília (DF); 2014. Available from: http://bvsms.saude.gov.br/bvs/publicacoes/vigitel_brasil_2013

f Instituto Brasileiro de Geografia e Estatística. Pesquisa Nacional de Saúde 2013: percepção do estado de saúde, estilos de vida e doenças crônicas - Brasil, Grandes Regiões e Unidades da Federação. Rio de Janeiro (RJ): IBGE; 2014. Available from: ftp://ftp.ibge.gov.br/PNS/2013/pns2013.pdf

g Ministério da Saúde, Secretaria de Atenção à Saúde, Departamento de Atenção Básica. Estratégias para o cuidado da pessoa com doença crônica: hipertensão arterial sistêmica. Brasília (DF); 2013. (Cadernos de Atenção Básica, 37). Available from: http://bvsms.saude.gov.br/bvs/publicacoes/estrategias_cuidado_pessoa_doenca_cronica.pdf

h Instituto Brasileiro de Geografia e Estatística. Pesquisa Nacional por Amostra de Domicílios - PNAD. Rio de Janeiro (RJ): IBGE; 2008. Available from: http://www.ibge.gov.br/home/estatistica/populacao/trabalhoerendimento/pnad2008/

i Ministério da Saúde, Secretaria de Ciência, Tecnologia e Insumos Estratégicos, Departamento de Assistência Farmacêutica e Insumos Estratégicos. Relação Nacional de Medicamentos Essenciais: RENAME 2013. 8 ed. Brasília (DF); 2013. Available from: http://www.sinfaerj.org.br/Arquivos/livro_rename_out_2013.pdf

## References

[1] Alleyne G, Binagwaho A, Haines A, Jahan S, Nugent R, Rojhani A (2013). Embedding non-communicable diseases in the post-2015 development agenda. Lancet.

[2] Andrade JP, Vilas-Boas F, Chagas H, Andrade M (2002). Epidemiological aspects of adherence to the treatment of hypertension. Arq Bras Cardiol.

[3] Atun R, Jaffar S, Nishtar S, Knaul FM, Barreto ML, Nyirenda M (2013). Improving responsiveness of health systems to non-communicable diseases. Lancet.

[4] Aziz MM, Calvo MC, Schneider IJC, Xavier AJ, d’Orsi E (2011). Prevalência e fatores associados ao acesso a medicamentos pela população idosa em uma capital do sul do Brasil: um estudo de base populacional. Cad Saude Publica.

[5] Barros MBA, Francisco PMSB, Zanchetta LM, César CLG (2011). Tendências das desigualdades sociais e demográficas na prevalência de doenças crônicas no Brasil, PNAD: 2003- 2008. Cienc Saude Coletiva.

[6] Beaglehole R, Bonita R, Horton R, Adams C, Alleyne G, Asaria P (2011). Priority actions for the non-communicable disease crisis. Lancet.

[7] Bertoldi AD, Barros AJD, Wagner A, Ross-Degnan D, Hallal PC (2009). Medicine access and utilization in a population covered by primary health care in Brazil. Health Policy.

[8] Turnbull F, Neal B, Ninomiya T, Algert C, Arima H, Blood Pressure Lowering Treatment Trialists Collaboration (2008). Effects of different regimens to lower blood pressure on major cardiovascular events in older and younger adults: meta-analysis of randomised trials. BMJ.

[9] Duarte MTC, Cyrino AP, Cerqueira ATAR, Nemes MIB, Iyda M (2010). Motivos do abandono do seguimento médico no cuidado a portadores de hipertensão arterial: a perspectiva do sujeito. Cienc Saude Coletiva.

[10] Falaschetti E, Mindell J, Knott C, Poulter N (2014). Hypertension management in England: a serial cross-sectional study from 1994 to 2011. Lancet.

[11] Ferreira RA, Barreto SM, Giatti L (2014). Hipertensão arterial referida e utilização de medicamentos de uso contínuo no Brasil: um estudo de base populacional. Cad Saude Publica.

[12] Fuchs FD, Gus M, Moreira WD, Moreira LB, Moraes RS, Rosito GA (1997). Blood pressure effects of antihypertensive drugs and changes in lifestyle in a Brazilian hypertensive cohort. J Hypertens.

[13] Gontijo MF, Ribeiro AQ, Klein CH, Rozenfeld S, Acurcio FA (2012). Uso de anti-hipertensivos e antidiabéticos por idosos: inquérito em Belo Horizonte, Minas Gerais, Brasil. Cad Saude Publica.

[14] Gu Q, Paulose-Ram R, Dillon C, Burt V (2006). Antihypertensive medication use among US adults with hypertension. Circulation.

[15] Gu Q, Burt VL, Dillon CF, Yoon S (2012). Trends in antihypertensive medication use and blood pressure control among United States adults with hypertension: the National Health and Nutrition Examination Survey, 2001 to 2010. Circulation.

[16] Joffres M, Falaschetti E, Gillespie C, Robitaille C, Loustalot F, Poulter N (2013). Hypertension prevalence, awareness, treatment and control in national surveys from England, the USA and Canada, and correlation with stroke and ischaemic heart disease mortality: a cross-sectional study. BMJ Open.

[17] Mengue SS, Bertoldi AD, Boing AC, Tavares NUL, Silva Dal Pizzol T da, Oliveira MA (2016). Pesquisa Nacional sobre Acesso, Utilização e Promoção do Uso Racional de Medicamentos (PNAUM): métodos do inquérito domiciliar. Rev Saude Publica.

[18] Ministério da Saúde, Secretaria de Políticas de Saúde (2000). Política Nacional de Medicamentos. Rev Saude Publica.

[19] Paniz VMV, Fassa AG, Facchini LA, Bertoldi AD, Piccini RX, Tomasi E (2008). Acesso a medicamentos de uso contínuo em adultos e idosos nas regiões Sul e Nordeste do Brasil. Cad Saude Publica.

[20] Pinho NA, Pierin AMG (2013). Hypertension control in Brazilian publications. Arq Bras Cardiol.

[21] Ruttanaumpawan P, Nopmaneejumruslers C, Logan AG, Lazarescu A, Qian I, Bradley TD (2009). Association between refractory hypertension and obstructive sleep apnea. J Hypertens.

[22] Schmidt MI, Duncan BB, Silva GA, Menezes AM, Monteiro CA, Barreto SM (2011). Chronic non-communicable diseases in Brazil: burden and current challenges. Lancet.

[23] Silva AL, Ribeiro AQ, Klein CH, Acurcio FA (2012). Utilização de medicamentos por idosos brasileiros, de acordo com a faixa etária: um inquérito postal. Cad Saude Publica.

[24] Sousa MR, Feitosa GS, Paola AA, Schneider JC, Feitosa-Filho GS, Nicolau JC (2011). I Diretriz da Sociedade Brasileira de Cardiologia sobre processos e competências para a formação em cardiologia no Brasil. Arq Bras Cardiol.

